# Yield of family screening for dilated cardiomyopathy: 10-year experience at a multidisciplinary cardiogenetic outpatient clinic

**DOI:** 10.1007/s12471-024-01924-1

**Published:** 2025-01-20

**Authors:** Isabelle P. Thierry, Steven A. Muller, Annette F. Baas, Dennis Dooijes, R. Laura E. van Loon, Angela E. Schoemaker, Pim van der Harst, Marish I. F. J. Oerlemans, Hubert F. Baars, Rutger J. Hassink, Folkert W. Asselbergs, J. Peter van Tintelen, Anneline S. J. M. te Riele

**Affiliations:** 1 Department of Cardiology, University Medical Centre Utrecht, Utrecht, The Netherlands; 2 Netherlands Heart Institute, Utrecht, The Netherlands; 3 Member of the European Reference Network for Rare and Low Prevalence Complex Diseases of the Heart: ERN GUARD-Heart’ (ERN GUARDHEART; http://guardheart.ern-net.eu), Utrecht, The Netherlands; 4 Department of Genetics, University Medical Centre Utrecht, Utrecht University, Utrecht, The Netherlands; 5 HartKliniek, Almere-Stad, The Netherlands; 6 Institute of Cardiovascular Science, Faculty Netherlands Health Sciences, University College London, London, UK; 7 Department of Cardiology, Amsterdam University Medical Centres, University of Amsterdam, Amsterdam, The Netherlands; 8 Health Data Research UK and Institute of Health Informatics, University College London, London, UK

**Keywords:** Cascade testing, Cascade screening, Cardiac screening, Cardiac testing, Family screening, Dilated cardiomyopathy, DCM

## Abstract

**Introduction:**

Current family screening approaches in dilated cardiomyopathy (DCM) depend on the presence or absence of a familial genetic variant, in which variant pathogenicity (i.e. benign or pathogenic) classification drives screening recommendations. However, this approach has never been systematically evaluated.

**Methods:**

To describe the yield of DCM family screening stratified by variant classification in the Netherlands, we included 358 relatives (mean age ± standard deviation: 44.4 ± 15.9 years at baseline; 52% female; 41% (likely) pathogenic (LP/P) variant carriers from 210 families). Demographics, symptoms and genetic/cardiac test results were obtained. Endpoints were the development of DCM (left ventricular ejection fraction < 50% of non-ischaemic aetiology) or occurrence of major adverse cardiovascular events (MACE) (i.e. heart failure hospitalisation, ventricular arrhythmia or death). Probability of DCM or MACE was assessed with the Kaplan-Meier method.

**Results:**

DCM was present in 32 relatives (9%) (25/32 (78%) with LP/P variant) at baseline and in an additional 10/97 relatives (10%) (9/10 (90%) with LP/P variant) who were re-evaluated during a median follow-up time of 5.0 years (interquartile range: 3.2–7.4). Of the 128 relatives without the familial LP/P variant, none developed DCM. MACE was experienced by 5 relatives (1%) (4/5 (80%) with LP/P variant), all of whom had DCM at the time of the event.

**Conclusion:**

The yield of DCM family screening was ~10% at baseline and another ~10% during 5‑year follow-up. Relatives without the familial LP/P variant could be safely discharged. These results reinforce the use of a genetics-first screening approach in relatives from families with an LP/P variant. This will lower the burden on resources in Dutch hospitals and help allocate resources to those who are most likely to benefit.

**Supplementary Information:**

The online version of this article (10.1007/s12471-024-01924-1) contains supplementary material, which is available to authorized users.

## What’s new?

- Relatives without a familial pathogenic or likely pathogenic variant did not have dilated cardiomyopathy (DCM) at baseline, nor did they develop DCM during ~ 5 years of follow-up.
- This study suggested the ‘genetic testing first’ approach, currently used for families with a pathogenic variant, can also be safely used in families with a likely pathogenic variant.

## Introduction

Dilated cardiomyopathy (DCM) is the most common cause of heart failure (HF), with an estimated prevalence of 1:250 individuals [1]. Although various aetiologies can lead to DCM, a genetic predisposition has been established in up to 46% of cases [2]. Establishing a genetic variant in the family enables cascade genetic testing in relatives, which can identify individuals at risk of DCM [3]. This means that cardiologists need to make management recommendations for an increasing number of genotype-positive relatives, many of whom are young and asymptomatic.

**Fig. 1:**
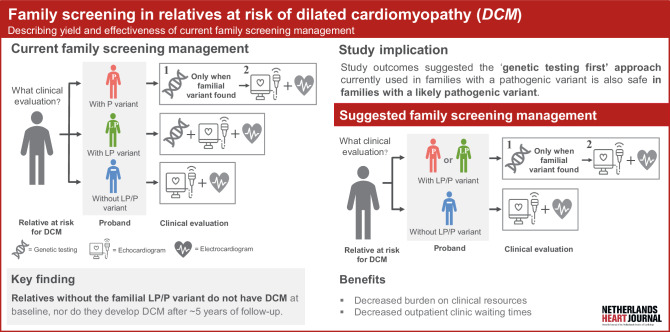
Infographic on family screening in relatives at risk of dilated cardiomyopathy (DCM)

Current guidelines for screening relatives at risk of DCM in the Netherlands rely on pathogenicity classification of the genetic variant in the family or proband [4]. In this context, genetic variants are classified as pathogenic (P) variant, likely pathogenic (LP) variant or variant of uncertain significance/no genetic variant found (‘gene-elusive’) [4]. This leads to 3 possible scenarios: (1) in case of a P variant in the family, all first-degree relatives of the proband are recommended to undergo genetic testing with subsequent cardiac testing only if they have the familial variant; (2) in case of an LP variant in the family, all first-degree relatives of the proband are recommended to undergo both cardiac and genetic testing at the time of the first evaluation; or (3) in case of a gene-elusive family, first-degree relatives are recommended to undergo cardiac testing without genetic testing. This Dutch approach is in contrast to the 2023 European Society of Cardiology (ESC) Guidelines for the management of cardiomyopathies, which recommend releasing LP variant carriers from cardiac testing if they do not have the familial variant (i.e. LP screening recommendations are identical to P screening recommendations) [3]. If this approach is confirmed to be safe, this would save many resources given the large number of LP variants identified. However, no research to date has systematically evaluated the yield of our Dutch DCM family screening approach. In this study, we aimed to describe the yield of DCM family screening stratified by classification of the familial genetic variant (Fig. 1).

## Methods

### Study population

For this retrospective study, we recruited relatives at risk of DCM visiting the dedicated cardiogenetic outpatient clinic at the Bergman Clinics in Bilthoven, the Netherlands for family screening between 2011 and 2022. All relatives underwent standardised evaluation, as described below, designed with close ties to the University Medical Centre Utrecht in Utrecht, the Netherlands. This study followed the Code of Conduct and the Use of Data in Health Research and was approved by the local ethics board (protocol number 19-115/C and UCC-UNRAVEL #12-387) [5].

### Clinical evaluation

Detailed clinical information regarding demographics, symptoms, cardiovascular risk factors and genetic/cardiac testing was obtained for every participant by review of medical records, clinical evaluation and patient history. Symptoms were divided into arrhythmic symptoms (composite of palpitations, presyncope and syncope), HF symptoms (composite of dyspnoea and oedema) and chest pain.

Pedigree analysis was performed by genetic counsellors or clinical geneticists with special interest in DCM. Relatives were divided based on their relationship to the proband as first- and second-degree relatives; first-degree relatives were additionally divided into parents, siblings and children of the proband. Variant adjudication was done by an expert laboratory geneticist (DD) based on the current consensus statement of the American College of Medical Genetics and Genomics [6]. Of note, in the period 2011–2022, different gene panels were used for genetic testing. Therefore, probands were considered as ‘gene-elusive’ if they were tested for all genes with definite, strong and moderate evidence for DCM [7] and proved not to have an LP/P variant. In case the proband was not tested for all genes with definite, strong and moderate evidence for DCM (and did not have an LP/P variant in the genes tested), the proband was considered as ‘genetic testing not performed’.

### Study outcomes

The primary endpoint was the development of DCM. Based on the most recent guidelines, [3, 8, 9] we used left ventricular ejection fraction (LVEF) < 50% to make the DCM diagnosis of non-ischaemic aetiology, as risk stratification and HF treatment are recommended to be initiated at this cut-off value.

The secondary endpoint was the occurrence of major adverse cardiovascular events (MACE), defined as hospitalisation for HF, occurrence of sustained ventricular tachycardia (lasting ≥ 30 s at ≥ 100 beats/min or requiring cardioversion), ventricular fibrillation/flutter or appropriate implantable-cardioverter defibrillator (ICD) intervention.

### Statistical analysis

Nominal variables are expressed as number (%) and continuous variables as mean ± standard deviation or median (interquartile range; IQR), as appropriate. Survival free from primary and secondary endpoints was visualised using Kaplan-Meier curves, and differences between variant classification were compared using the log-rank test. A *p*-value < 0.05 was considered to be statistically significant. Statistical analyses were performed using IBM SPSS Statistics for Windows, version 29.0, and R version 4.1.2 (Boston, MA, USA).

## Results

### Study population

Our study cohort consisted of 358 relatives from 210 families. Their baseline characteristics are shown in Tab. 1. Mean age at the first evaluation was 44.4 ± 15.9 years, and 185 (52%) were female. Most relatives (*n* = 238; 66%) were first-degree relative of the proband. Of the remaining 120 subjects (34%) who were second-degree relatives of the proband, 109 (91%) were from a family with a causative LP/P DCM variant, whereas the remaining 11 (9%) had their own first-degree relative with a DCM or sudden cardiac death diagnosis and hence an indication for screening.

**Table 1:** Baseline characteristics of study population

			Phenotype of relative at baseline			
			Study cohort (*N* = 358)	No DCM (*n* = 326)	DCM diagnosis (*n* = 32)	*P*-value
*Demographic data*						
Female			185 (52)	167 (51)	18 (56)	0.587
Age at baseline, years			44.4 ± 15.9	43.9 ± 15.8	50.3 ± 16.7	0.023
Follow-up time, years			5.0 (3.2–7.4)	5.0 (3.3–7.6)	5.9 (3.2–7.0)	0.312
*Relationship to proband*						0.003
Sibling			82 (23)	68 (21)	14 (44)	
Child			38 (11)	32 (10)	6 (19)	
Parent			118 (33)	110 (34)	8 (25)	
≥ Second-degree family member			120 (34)	116 (36)	4 (13)	
*Genotype of relative*						< 0.001
*P* variant carrier			49 (14)	40 (12)	9 (28)	
LP variant carrier			98 (27)	82 (25)	16 (50)	
Not harbouring familial variant			128 (36)	128 (39)	0 (0)	
Gene-elusive proband			22 (6)	18 (6)	4 (13)	
Genetic testing not performed			61 (17)	58 (18)	3 (9)	
G + gene variants found in relative^a^						0.023
*TTN*			82 (23)	68 (21)	14 (44)	
*LMNA*			9 (3)	9 (3)	0 (0)	
*PLN*			8 (2)	7 (2)	1 (3)	
*FLNC*			10 (3)	10 (3)	0 (0)	
*MYH7*			6 (2)	4 (1)	2 (6)	
Other variants			28 (8)	23 (7)	5 (16)	
> 1 variant			4 (1)	1 (0)	3 (9)	
*Symptoms at baseline*^*b*^			110 (31)	90 (28)	20 (63)	< 0.001
Arrhythmic symptoms			35 (10)	30 (9)	5 (16)	0.379
Heart failure symptoms			38 (11)	29 (9)	9 (28)	0.004
Chest pain			41 (11)	32 (10)	9 (28)	0.007
*Risk factors*						
Smoking						0.468
Current			33 (9)	28 (9)	5 (16)	
Past			26 (7)	23 (7)	3 (9)	
Never			146 (41)	136 (42)	10 (31)	
Hypercholesterolaemia			63 (18)	56 (17)	7 (22)	0.871
Hypertension			174 (49)	160 (49)	14 (44)	0.964
Diabetes			157 (44)	139 (43)	18 (56)	0.731

Data are *n* (%), mean ± standard deviation or median (interquartile range)

^a^Five most frequently found G + variants in relatives. Other variants are visualised in Figure S1 in the Electronic Supplementary Material. When relative had > 1 G + variant, this is defined as > 1 variant

^b^Arrhythmic symptoms are defined as syncope, presyncope and palpitations; heart failure symptoms are defined as dyspnoea and oedema

*P* pathogenic, *LP* likely pathogenic, *TTN* titin, *LMNA* lamin A/C, *PLN* phospholamban, *FLNC* filamin C, *MYH7* beta-myosin heavy-chain

Genotype distribution is shown in Figure S1 in the Electronic Supplementary Material. An LP/P variant was found in 147 of the 358 relatives (41%), with titin (*TTN*) being the most commonly affected gene (82/147; 56%). Of the 128 relatives without a familial LP/P variant, 98 (77%) were relatives of a proband with an LP variant. Overall, most relatives were asymptomatic and came to attention because of screening (*n* = 248; 69%), whereas the remaining 110 relatives (31%) reported arrhythmic symptoms (*n* = 35/110; 32%), HF symptoms (*n* = 38/110; 35%) and chest pain (*n* = 41/110; 37%) at a comparable rate.

### First clinical evaluation

**Fig. 2:**
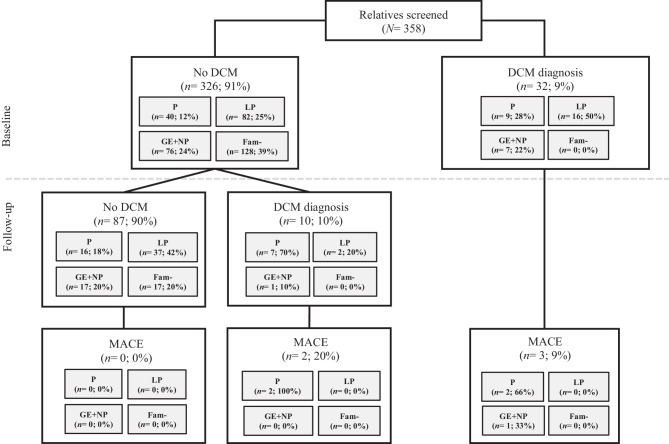
Flowchart of study cohort showing relatives diagnosed with or without dilated cardiomyopathy (*DCM*) at baseline and follow-up. Numbers of relatives with major adverse cardiovascular events (*MACE*) are shown in the bottom-most boxes. *P* pathogenic variant carrier, *LP *likely pathogenic variant carrier, *GE* *+* *NP *relatives from gene-elusive families without LP/P variant or in whom no genetic testing was performed, *Fam- *relatives without familial variant

Figure 2 depicts the flowchart of the study population. At the first evaluation, 32 relatives (9%) were diagnosed with DCM. As shown in Tab. 1, relatives with DCM at the first evaluation were significantly older than those without a DCM diagnosis (mean age: 50.3 ± 16.7 years vs 43.9 ± 15.8 years; *p* = 0.023), whereas sex was equally distributed between the 2 groups (females: 56% vs 51%; *p* = 0.587). Additionally, relatives with DCM were significantly more likely to be symptomatic (20 (63%) vs 90 (28%); *p* < 0.001) and harbour the familial LP/P variant (25 (78%) vs 125 (37%); *p* < 0.001) (Tab. 1). Consequently, the yield of screening for DCM in symptomatic relatives was significantly higher (*n* = 20/110; 18%) compared with asymptomatic relatives (*n* = 12/248; 5%; *p* < 0.001). Of note, 2/32 relatives (6%) who were diagnosed with DCM at baseline had experienced a previous HF hospitalisation (Tab. 2), stressing the importance of timely initiation of family screening.

**Table 2:** Characteristics of relatives with *MACE*

Relative	Sex	Phenotype at baseline	Age at MACE, years	Variant classification and gene	MACE	Time from first visit until MACE, years	Time from diagnosis until MACE, years	Cardiovascular co-morbidities	Symptomatic at baseline
1	F	Diagnosed with DCM	53.6	*P*; *TTN*	HF	0	0	Smoking: current	Yes, heart failure symptoms
								HT: no	
								HC: no	
								Diabetes: no	
2	F	Diagnosed with DCM	53.5	*P*; *TTN*	HF	0	0	Smoking: never	Yes, heart failure symptoms
								HT: no	
								HC: no	
								Diabetes: no	
3	F	No DCM	45.7	*P*; *TNNI3*	OHCA VF	5.8	0	Smoking: past	Yes, chest pain
								HT: no	
								HC: yes	
								Diabetes: no	
4	M	No DCM	65.9	*P*; *TNNI3*	VT	4.4	1.3	Smoking: past	No
								HT: no	
								HC: no	
								Diabetes: no	
5	M	Diagnosed with DCM	55.6	GE	OHCA VF	5.7	5.7	Smoking: never	No
								HT: yes	
								HC: no	
								Diabetes: no	

*MACE* major adverse cardiovascular events, *P* pathogenic, *TTN* titin, *TNNI3* troponin I3, *GE* gene-elusive, *HF* heart failure, *OHCA VF* out-of-hospital cardiac arrest based on ventricular fibrillation, *VT* ventricular tachycardia, *HT* hypertension, *HC* hypercholesterolaemia

**Fig. 3:**
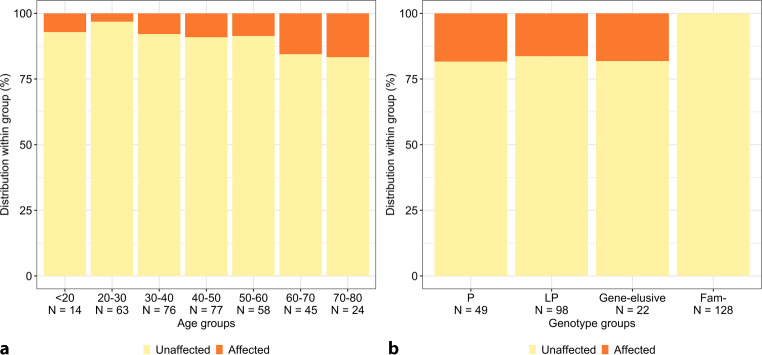
Yield of screening. Distribution of relatives diagnosed with dilated cardiomyopathy (*DCM*; orange) and without DCM (yellow) stratified by (**a**) age and (**b**) genotype. Numbers at risk are shown below each bar. *Fam- *relatives without familial variant

Figure 3a visualises the yield of screening stratified by age. Of note, we diagnosed 1 relative with DCM at 4 years of age. This relative was first evaluated at the age of 4 years after her sister (proband) died of DCM at the age of 8 months. Further family screening identified DCM in both her father and brother. Genetic testing remained negative in this family, despite extensive whole-exome sequencing in multiple family members diagnosed with DCM.

### Genotype-specific yield of cardiac screening at baseline

Figure 3b shows the yield of screening stratified by genetic testing results. As shown, relatives with the P variant (*n* = 9/49; 18%) or LP variant (*n* = 16/98; 16%) or those from a gene-elusive family (*n* = 4/22; 18%) had comparable rates of DCM diagnosis at the first evaluation. In contrast, none of the 128 relatives without the familial LP/P variant had DCM at the baseline evaluation (0%; *p* < 0.001).

### Progression towards dilated cardiomyopathy

Among 326 relatives without DCM at the first evaluation, 97 (30%) had ≥ 1 follow-up evaluation (Fig. 2, and see Table S1 in Electronic Supplementary Material). As expected, relatives who had undergone genetic testing and proved to be negative for the familial LP/P variant were more likely to have no follow-up evaluation. In contrast, those who had HF symptoms were more likely to have follow-up data available. Relatives without the familial variant who were followed up underwent genetic testing at a follow-up evaluation.

**Fig. 4:**
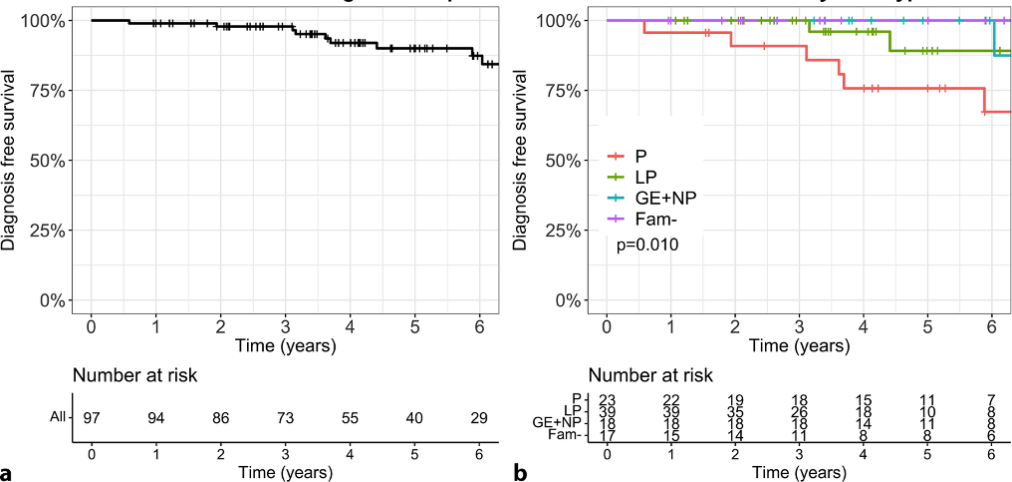
Penetrance of dilated cardiomyopathy (DCM) during follow-up. Survival curve of DCM diagnosis in (**a**) overall study cohort and (**b**) stratified by genotype. Relatives with pathogenic (P) or likely pathogenic (LP) variant are visualised by red and green lines, respectively. Relatives from gene-elusive families without LP/P variant or in whom no genetic testing was performed (GE + NP) are depicted by blue line. Relatives without familial variant (*Fam-*) are visualised by purple line

These 97 relatives were followed for a median duration of 4.9 years (IQR: 3.3–7.4). Overall, 10 relatives (10%) progressed to DCM, with a median time to DCM diagnosis of 3.7 years (IQR: 3.1–5.5) (Fig. 4a). As shown in Table S2 in the Electronic Supplementary Material, relatives who progressed to DCM were more likely to be a sibling of the proband (4 (40%) vs 18 (21%); *p* = 0.038) and had a longer follow-up time (6.4 years; IQR: 5.5–8.8 vs 4.2 years; IQR: 3.0–7.1; *p* = 0.026) compared with those without DCM during follow-up.

### Genotype-specific yield of cardiac screening during follow-up

Figure 4b shows the progression towards DCM stratified by genotype during follow-up. Relatives carrying the familial P variant progressed fastest to DCM (7/23 relatives; median time to diagnosis 3.6 years; IQR: 2.5–4.8), followed by relatives carrying the familial LP variant (2/39 relatives; time to diagnosis 3.6 and 4.4 years, respectively) and relatives from gene-elusive families (1/18 relatives; time to diagnosis: 6.0 years; *p* = 0.010). Relatives without the familial LP/P variant did not develop DCM during follow-up. These relatives had had genetic testing done at a follow-up evaluation.

### Occurrence of major adverse cardiovascular events

As shown in Tab. 2, 5 of the 358 relatives (1%) experienced MACE. As previously mentioned, MACE already occurred in 2 relatives (40%) prior to their first evaluation (relatives 1 and 2 in Tab. 2): they had been previously hospitalised for HF and were proven carriers of the familial *P TTN* variant. Relative 3 in Tab. 2 carried a P troponin I3 (*TNNI3*) variant, was not diagnosed with DCM at baseline and subsequently withdrew from follow-up evaluation. After 5.8 years, she presented with an out-of-hospital cardiac arrest. During the subsequent hospitalisation, an echocardiogram showed an LVEF of 28%. Her relative (relative 4 in Tab. 2) carried the same variant and was not diagnosed with DCM at baseline. During follow-up evaluation, he progressed to DCM and experienced ventricular tachycardia (cycle length: 353 ms) 1.3 years after DCM diagnosis. Subsequently, he received an ICD for secondary prevention. Relative 5 was diagnosed with DCM at baseline (LVEF 45%). During follow-up, he had an out-of-hospital cardiac arrest from which he was successfully resuscitated.

## Discussion

The genetic era has led to an increasing number of relatives at risk of DCM coming to clinical attention. To the best of our knowledge, this study is the first to evaluate the yield of clinical DCM diagnosis and the possible optimisation of family screening for DCM in the Netherlands.

This study has several interesting results. First, the yield of screening at both baseline and after 5 years of follow-up was ~10%. Additionally, an age-related penetrance of DCM was observed. The yield was notably low in those < 20 years of age, with only 1 relative diagnosed with DCM who also had a proband diagnosed at infancy. This is in line with the results of a previous study [10] and implies that screening of relatives may be initiated at > 20 years of age unless the proband in the family was diagnosed before or during adolescence or an LP/P variant with known paediatric DCM onset is identified in the family [11].

Second, relatives without the LP/P familial variant did not have DCM at baseline, nor progression towards DCM during follow-up. These results are in line with a recent Danish study [12] and add to the body of evidence that relatives from LP families should first be offered genetic testing and can safely be discharged from cardiac screening when genetic testing proves to be negative (unless they are symptomatic). This also suggests that the 2023 ESC Guidelines for the management of cardiomyopathies may be safely implemented in the Netherlands.

Third, the baseline yield of DCM diagnosis in relatives of gene-elusive probands was unexpectedly high and comparable to those carrying an LP/P variant. As some of our cohort participants were relatives who received their genetic testing results more than a decade ago, this likely included probands who did not undergo genetic testing with the comprehensive panels and techniques we are currently offering. It is well known that updating genetic testing in the proband is beneficial in identifying the familial genetic variant [13, 14]. We therefore deem it likely that repeated genetic testing (which was not available for this study), would have identified an LP/P variant in a proportion of these families. This again reinforces the importance of updating genetic testing in DCM evaluation and shows that gene-elusive families may not be considered as gene-elusive in the future.

Last, our study shows that adherence to family screening ensures the diagnosis of DCM in all relatives prior to the occurrence of MACE, which is in line with previous reports (see Table S3 in Electronic Supplementary Material [10, 12, 15–19]) and family screening recommendations for other cardiomyopathies [3, 20–25]. Although this is reassuring, there is an increasing body of evidence suggesting that the clinical course of DCM is gene-specific, suggesting that gene-specific family screening algorithms may be beneficial [11, 26–30].

### Study limitations and future perspectives

In this study population, we cannot exclude selection bias. For example, only 8 relatives carried the phospholamban (*PLN*) p.Arg14del variant, whereas a higher incidence was expected considering it is a Dutch founder variant. This could be explained by direct referral of *PLN* relatives to the University Medical Centre Utrecht rather than the Bergman Clinics because of its arrhythmogenic nature [27].

Next, the family size of relatives in our study ranged from 1 to 11 subjects. These large families could have skewed our results. Additionally, the study population consisted of only 14 relatives younger than 20 years since the Bergman Clinics did not routinely perform screening in paediatric subjects. Furthermore, we cannot rule out that relatives who were older and/or symptomatic were more likely to present for family screening, thereby leading to selection bias.

Moreover, the number of relatives from gene-elusive probands in our study was low, and the yield of screening in these individuals should be interpreted with caution. Last, it would have been of interest to investigate the gene-specific yield of family screening, as it is increasingly recognised that the clinical disease course is gene-specific. It will require large multicentre efforts to collect enough variant carriers per gene for meaningful analyses.

## Conclusion

We evaluated the yield of family screening in relatives at risk of developing DCM. We showed that the ‘genetic testing first’ approach currently used for families with a P variant is also good clinical practice for families with an LP variant, as proposed in the 2023 ESC Guidelines for the management of cardiomyopathies. This will lower the burden on resources in Dutch outpatient clinics and hospitals and help allocate resources to those who are most likely to benefit from cardiac care.

## Supplementary Information

The Supplementary Information includes detailed tables on the characteristics of unaffected relatives and studies on family screening for DCM, along with a figure visualising the genotype distribution of the study population.
