# Supplementary material for: Yield of family screening for dilated cardiomyopathy: 10-year experience at a multidisciplinary cardiogenetic outpatient clinic
Source: Neth Heart J. 2025 Jan 20;33(2):46–54. doi: 10.1007/s12471-024-01924-1 (PMC11757811; doi:10.1007/s12471-024-01924-1)
Supplement: Supplementary file 1 — The Supplementary Information includes detailed tables on the characteristics of unaffected relatives and studies on family screening for DCM, along with a figure visualising the genotype distribution of the study population. [file 12471_2024_1924_MOESM1_ESM.docx]

**Table S1** Characteristics of relatives unaffected at baseline with 1 evaluation or follow-up

|  | Relatives Unaffected at Baseline | | |  |
| --- | --- | --- | --- | --- |
|  |  |  |  |  |
|  | Total  (n = 326) | With one evaluation  (n = 229) | With FU  (n = 97) | P-value |
| Demographic data |  |  |  |  |
| Female sex | 166 (51) | 116 (51) | 50 (52) | 0.843 |
| Age at baseline (y) | 43.9±15.8 | 43.8±15.7 | 44.1±16.0 | 0.855 |
| Relationship to proband |  |  |  | 0.082 |
| Sibling | 68 (21) | 46 (20) | 22 (23) |  |
| Child | 31 (10) | 22 (10) | 9 (9) |  |
| Parent | 109 (33) | 70 (31) | 39 (40) |  |
| ≥2-degree family member | 112 (34) | 89 (39) | 23 (24) |  |
| Genotype relative |  |  |  | <0.001 |
| Class 5 carrier | 40 (12) | 17 (7) | 23 (24) |  |
| Class 4 carrier | 82 (25) | 43 (19) | 39 (40) |  |
| Not harbouring familial variant | 128 (39) | 111 (49) | 17 (18) |  |
| Gene elusive | 18 (6) | 15 (7) | 3 (3) |  |
| Genetic testing not performed | 58 (18) | 43 (19) | 15 (16) |  |
| G+ gene variants found in relative^1^ |  |  |  | 0.044 |
| *TTN* | 68 (21) | 39 (17) | 29 (30) |  |
| *LMNA* | 9 (3) | 2 (1) | 7 (7) |  |
| *PLN* | 7 (2) | 3 (1) | 4 (4) |  |
| *FLNC* | 10 (3) | 6 (3) | 4 (4) |  |
| *MYH7* | 4 (1) | 0 (0) | 4 (4) |  |
| Other variant | 23 (7) | 9 (4) | 14 (14) |  |
| >1 variant | 1 (0) | 0 (0) | 1 (1) |  |
| Symptoms at baseline^2^ | 89 (27) | 57 (25) | 32 (33) | 0.125 |
| Arrhythmic symptoms | 30 (9) | 19 (8) | 11 (11) | 0.449 |
| Heart failure symptoms | 28 (9) | 13 (6) | 15 (16) | 0.004 |
| Chest pain | 31 (10) | 25 (11) | 6 (6) | 0.186 |
| Risk factors |  |  |  |  |
| Smoker |  |  |  | 0.129 |
| Current | 28 (9) | 17 (7) | 11 (11) |  |
| Past | 23 (7) | 12 (5) | 11 (11) |  |
| Never | 134 (41) | 96 (42) | 38 (39) |  |
| Hypercholesterolemia | 27 (8) | 20 (9) | 7 (7) | 0.893 |
| Hypertension | 59 (18) | 36 (16) | 23 (24) | 0.088 |
| Diabetes | 11 (3) | 7 (3) | 4 (4) | 0.566 |

Data are expressed as mean±standard deviation, median [interquartile range], or as number (%) as appropriate.

*TTN* = titin; *LMNA* = lamin A/C; *PLN* = phospholamban; *FLNC* = filamin C; *MYH7* = beta-myosin heavy-chain. ^1^The five most frequently found G+ variants in the relatives. Other variants are visualized in **Fig. S1** in the Electronic Supplementary Material. When more than one G+ variant was present in the relative, it is defined as >1 variant. ^2^Arrhythmic symptoms are defined as syncope, presyncope, and palpitations; heart failure symptoms are defined as dyspnoea and oedema

**Table S2** Characteristics of relatives unaffected at baseline with follow-up

|  | Relatives Unaffected at Baseline with Follow-Up | | |  |
| --- | --- | --- | --- | --- |
|  |  |  |  |  |
|  | Total  (n = 97) | Remaining unaffected  (n = 87) | Developing DCM  (n = 10) | P-value |
| Demographic data |  |  |  |  |
| Female sex | 51 (53) | 46 (53) | 5 (50) | 0.863 |
| Age at baseline (y) | 44.1±16.0 | 43.7±16.0 | 47.1±16.4 | 0.488 |
| FU time (y) | 4.9 [3.3 - 7.4] | 4.2 [3.0 - 7.1] | 6.4 [5.5 - 8.8] | 0.024 |
| Relationship to proband |  |  |  | 0.040 |
| Sibling | 22 (23) | 18 (21) | 4 (40) |  |
| Child | 9 (9) | 9 (10) | 0 (0) |  |
| Parent | 40 (41) | 35 (40) | 5 (50) |  |
| ≥2-degree family member | 23 (24) | 23 (26) | 0 (0) |  |
| Genotype relative |  |  |  | 0.002 |
| P variant carrier | 23 (24) | 16 (18) | 7 (70) |  |
| LP variant carrier | 39 (40) | 37 (43) | 2 (20) |  |
| Not harbouring familial variant | 17 (18) | 17 (20) | 0 (0) |  |
| Gene elusive | 3 (3) | 2 (2) | 1 (10) |  |
| Genetic testing not performed | 15 (15) | 15 (17) | 0 (0) |  |
| G+ gene variants found in relative^1^ |  |  |  | 0.001 |
| *TTN* | 29 (30) | 25 (29) | 4 (40) |  |
| *LMNA* | 7 (7) | 7 (8) | 0 (0) |  |
| *PLN* | 4 (4) | 3 (3) | 1 (10) |  |
| *FLNC* | 4 (4) | 4 (5) | 0 (0) |  |
| *MYH7* | 4 (4) | 3 (3) | 1 (10) |  |
| *TNNI3* | 3 (3) | 0 (0) | 3 (30) |  |
| Other variant | 11 (11) | 11 (13) | 0 (0) |  |
| Symptoms at baseline^2^ | 33 (34) | 28 (32) | 5 (50) | 0.294 |
| Arrhythmic symptoms | 11 (11) | 10 (12) | 1 (10) | 0.750 |
| Heart failure symptoms | 16 (16) | 14 (X) | 2 (20) | 0.782 |
| Chest pain | 6 (6) | 3 (3) | 3 (30) | 0.011 |
| Risk factors |  |  |  |  |
| Smoker |  |  |  | 0.855 |
| Current | 11 (11) | 9 (10) | 2 (20) |  |
| Past | 11 (11) | 9 (10) | 2 (20) |  |
| Never | 39 (40) | 34 (39) | 5 (50) |  |
| Hypercholesterolemia | 7 (7) | 5 (6) | 2 (20) | 0.720 |
| Hypertension | 24 (25) | 22 (25) | 2 (20) | 0.345 |
| Diabetes | 4 (4) | 4 (5) | 0 (0) | 0.221 |

Data are expressed as mean±standard deviation, median [interquartile range], or as number (%) as appropriate.

FU = follow-up; P = pathogenic; LP = likely pathogenic; *TNNI3* = Troponin I; *TTN* = titin; *LMNA* = lamin A/C; *PLN* = phospholamban; *FLNC* = filamin C; *MYH7* = beta-myosin heavy-chain. ^1^The five most frequently found G+ variants in the relatives. Other variants are visualized in **Fig. S1** in the Electronic Supplementary Material. When more than one G+ variant was present in the relative, it is defined as >1 variant. ^2^Arrhythmic symptoms are defined as syncope, presyncope, and palpitations; heart failure symptoms are defined as dyspnoea and oedema

**Table S3** Studies performed on DCM family screening

| **First author, publication year** | **Study region/**  **Cohort** | **Study design** | **DCM definition** | **Sample size** | **Age at first evaluation** | **Genotype** | **Baseline yield of screening** | **Duration of follow-up (if present)** | **Yield of serial evaluation** |
| --- | --- | --- | --- | --- | --- | --- | --- | --- | --- |
| Gimeno, 2009 | Murcia, Spain | Retrospective study from tertiary center | LVFS<25% and LVEDD≧112% | 378 | Not specified | Unknown | 16% | NA |  |
| Vissing, 2022 | Copenhagen, Denmark | Retrospective cohort study from tertiary center | LVEF<45% and LVEDD>2SD | 563 | 38±17 | Genotype + and genotype - | 22% | 5.3 (IQR 2.7-8.8) | 10% |
| Michels, 1992 | Rochester, USA | Prospective cohort study from tertiary center | LVEF<50% and LVEDD>95^th^ percentile adjusted for age and BSA | 322 | 38 (range 1 day to 84.1 years) | Unknown | 5% | NA | NA |
| Michels, 2003 | Rochester, USA | Retrospective cohort study from tertiary center | LVEF<50% and LVEDD>95^th^ percentile adjusted for age and BSA | 130 (only reported FU as baseline is same as Michels 1999) | Not specified | Unknown | NA | 10.5 (IQR 8-12) | 9% |
| Huggins, 2022 | USA, DCM precision consortium | Prospective cohort study from mostly tertiary centers | LVEF<50% and LVEDD >95th percentile adjusted age and sex " | 1693 | Depending on group between 28.4 (21.1-36.7), and 44.5 (28.1-60.1) | Unknown | 9% | NA | NA |
| Fudikara, 2023 | Tanzania | Retrospective cohort study from tertiary center | LVEF<50% and LVEDV-I >112% | 120 | 39.0 (29.3-49.0) | Unknown | 3% | NA | NA |
| Hey, 2020 | Odense, Denmark | Retrospective cohort study from tertiary center | LVEF<45% and LVEDD>112% | 433 | Not specified | Unknown | 18% | NA | NA |

**Figure S1** Genotype distribution in the study population


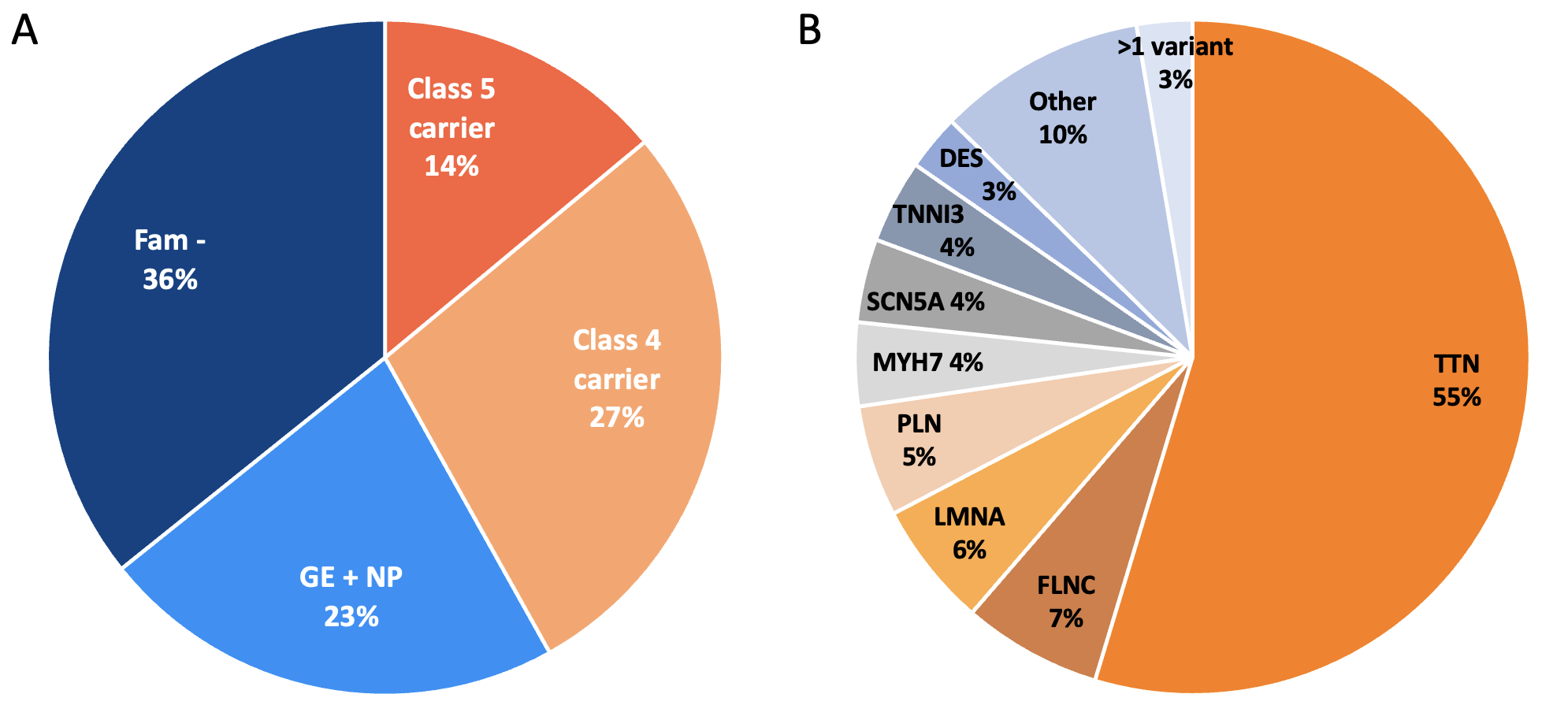


Panel A: Distribution of genotype in study population stratified by class 5 carriers (red), class 4 carriers (orange), gene elusive relatives and genetic testing not performed (GE+NP; light blue), and relatives not harbouring the familial variant (Fam-; dark blue). Panel B: Distribution of (likely) pathogenic genes found in relatives. *TTN* = titin; *FLNC* = filamin C; *LMNA* = lamin A/C; *PLN* = phospholamban; *MYH7* = beta-myosin heavy-chain; *SCN5A* = sodium voltage-gated channel alpha subunit 5; *TNNI3* = troponin I3; *DES* = desmin
